# Factors associated with severe maternal outcome in patients admitted to an intensive care unit in northeastern Brazil with postpartum hemorrhage: a retrospective cohort study

**DOI:** 10.1186/s12884-023-05874-1

**Published:** 2023-08-10

**Authors:** André Vieira Lanza, Melania Maria Amorim, Mayara Ferreira, Claúdia Menezes Cavalcante, Leila Katz

**Affiliations:** 1 Teaching Hospital of the Federal University of Uberlândia (UFU), Minas Gerais Uberlândia, Brazil; 2 Instituto de Medicina Integral Prof. Fernando Figueira (IMIP), Recife, Pernambuco, Brazil; 3 Federal University of Campina Grande (UFCG), Campina Grande, Paraíba, Brazil; 4 IMIP, Recife, Pernambuco Brazil; 5 Faculdade Pernambucana de Saúde (FPS), Recife, Pernambuco Brazil; 6 Recife, Brazil

**Keywords:** Postpartum hemorrhage, Third stage of labor, Maternal death, Complications of labor, Pregnancy

## Abstract

**Background:**

Postpartum hemorrhage (PPH) is the leading cause of maternal death worldwide, particularly in low- and middle-income countries; however, the majority of these deaths could be avoided with adequate obstetric care. Analyzing severe maternal outcomes (SMO) has been a major approach for evaluating the quality of the obstetric care provided, since the morbid events that lead to maternal death generally occur in sequence. The objective of this study was to analyze the clinical profile, management, maternal outcomes and factors associated with SMO in women who developed PPH and were admitted to an obstetric intensive care unit (ICU) in northeastern Brazil.

**Methods:**

This retrospective cohort study included a non-probabilistic, consecutive sample of postpartum women with a diagnosis of PPH who were admitted to the obstetric ICU of the *Instituto de Medicina Integral Prof. Fernando Figueira* (IMIP) between January 2012 and March 2020. Sociodemographic, biological and obstetric characteristics and data regarding childbirth, the management of PPH and outcomes were collected and analyzed. The frequency of maternal near miss (MNM) and death was calculated. Multiple logistic regression analysis was performed to determine the adjusted odd ratios (AOR) and their 95% confidence intervals (95% CI) for a SMO.

**Results:**

Overall, 136 cases of SMO were identified (37.9%), with 125 cases of MNM (34.9%) and 11 cases of maternal death (3.0%). The factors that remained associated with an SMO following multivariate analysis were gestational age ≤ 34 weeks (AOR = 2.01; 95% CI: 1.12–3.64; *p* < 0.02), multiparity (AOR = 2.20; 95% CI: 1.10–4.68; *p* = 0.02) and not having delivered in the institute (AOR = 2.22; 955 CI: 1.02–4.81; *p* = 0.04).

**Conclusion:**

Women admitted to the obstetric ICU with a diagnosis of PPH who had had two or more previous deliveries, gestational age ≤ 34 weeks and who had delivered elsewhere were more likely to have a SMO.

**Supplementary Information:**

The online version contains supplementary material available at 10.1186/s12884-023-05874-1.

## Introduction

Around 830 women die every day worldwide from complications related to pregnancy and the postpartum period. Low- and middle-income countries account for 99% of these deaths, with an estimated risk of death of 1 in 41 compared to 1 in 3,300 in high-income countries [1]. Many deaths could be avoided if adequate obstetric care were provided.

The rate of postpartum hemorrhage (PPH) has increased with changes in obstetric practice and is associated with maternal obesity, placenta previa, preterm delivery, transverse position, macrosomia, uterine fibroids and preeclampsia, as well as with ethnicity/skin color [2, 3]. In patients who suffer a hemorrhage, these factors may also predict prognosis; however, the weight of the maternal characteristics appears to fade compared to the importance of prophylaxis and the appropriate management of PPH [2].

Active management of the third stage of labor is an effective means of preventing PPH, particularly the prophylactic use of uterotonics shortly after childbirth [4]. Since uterine atony is the main cause of PPH, treatment consists of administering multiple uterotonic agents in quick succession, together with tranexamic acid, a drug shown to be capable of reducing obstetric mortality [5]. When ineffective, other interventions then follow in quick succession [6–9]. The hemorrhage should be controlled within a circuit of pharmacological and surgical procedures before resorting to hysterectomy and, during this process, calling for help and involving trained personnel is crucial [10].

In 2008, the World Health Organization (WHO) adopted a list of 25 criteria for the identification of a maternal near miss (MNM), including: cardiovascular dysfunction/signs of shock, renal dysfunction, respiratory dysfunction, coagulation dysfunction, hepatic dysfunction, neurological dysfunction and uterine dysfunction leading to hysterectomy [11]. Cases of MNM, together with cases of maternal death, make up what is referred to as severe maternal outcomes (SMOs). Maternal death should be a rare event; therefore, the set of SMOs is currently assessed to strategically evaluate the quality of the obstetric care provided, since the events that lead to maternal death tend to occur in sequence. The quicker a potentially fatal condition is identified, the lower the risk of death or of permanent sequelae [12–14].

The “*Childbirth in Brazil*” study was the first nationwide, epidemiological study to describe healthcare provided during labor and childbirth. Overall, 23,984 women and their babies were included in the study between February 2011 and October 2012 [15, 16]. The incidence of MNM was 10.21 per 1,000 liveborn infants, with an MNM ratio of 30.8 for each maternal death. Although that survey did not focus on the causes of maternal morbidity, PPH is known to be one of the principal causes of maternal death in the country [15, 17].

Analyzing the patterns of maternal mortality and morbidity and the occurrence of SMO cases in a population can help in the management of resources and care that is needed to improve maternal and child healthcare. This can interrupt the sequence of events that results in maternal death, particularly from avoidable causes such as PPH [14].

The present study was conducted to analyze the clinical profile, maternal outcomes and, principally, the factors associated with SMO in women who developed PPH and were admitted to the obstetric intensive care unit (ICU) at the *Instituto de Medicina Integral Prof. Fernando Figueira* (IMIP).

## Methods

This retrospective cohort study was conducted in the obstetric ICU at IMIP, a referral center for the care of pregnant women at increased maternal and neonatal risk in Recife, Pernambuco, Brazil. Women diagnosed with PPH who had been admitted to the ICU between January 2012 and March 2020 were evaluated. IMIP’s Ethics Committee approved the study protocol under reference CAAE 37124820.2.0000.5201 and the requirement for informed consent was waived. The STROBE guidelines for reporting cohort studies were followed.

A non-probabilistic, consecutive sample was obtained from all the available hospital records that met the inclusion criteria (postpartum women admitted to IMIP’s obstetric ICU due to hemorrhage between January 2012 and March 2020). The records were evaluated and the data of interest extracted. Patients whose diagnosis of hemorrhage or postpartum hemorrhage was ruled out after their hospital records were examined were excluded from the study.

*Postpartum hemorrhage/PPH* was defined as a cumulative loss of blood ≥ 1000 ml or bleeding associated with signs and symptoms of hypovolemia up to 24 h following childbirth. Of note, PPH was previously defined as a loss of ≥ 500 ml of blood following vaginal delivery or ≥ 1000 ml of blood following Cesarean section [10].

*Management of the third stage of labor* was defined as a set of management strategies that could be implemented shortly after the expulsive period as prophylaxis against PPH. These strategies are separated into: expectant management (wait for the spontaneous delivery of the placenta), active management (use of uterotonics, clamping of the umbilical cord and controlled cord traction) or mixed management (use of some but not all the forms of active management) [10].

*Maternal near miss/MNM* refers to a very ill pregnant or recently delivered woman who nearly died but survived a complication during pregnancy, childbirth or within 42 days of termination of pregnancy according to the presence of at least one of the criteria adopted by the WHO. *Maternal death* is death occurring during pregnancy or within 42 days of termination of pregnancy excluding accidental or incidental causes. *Severe maternal outcome/SMO* refers to the sum of the cases of maternal death and maternal near miss [18].

### Statistical analysis

Epi Info, version 7.2.5.0 and Medcalc, version 11.112 were used throughout the analysis. Contingency tables were constructed and the chi-square test of association and Fisher’s exact test were used, as appropriate. Risk ratios (RR) and their 95% confidence intervals (95% CI) were calculated to determine the association between the independent or predictive variables and SMO. The standard risk of 1.0 was attributed to the reference category. All *p*-values were two-tailed and the significance level was fixed at 5%.

A hierarchical logistic regression model was used in which the variables were entered in accordance with the significance level and were divided into blocks according to their proximity to the outcome. In each block, the variables that remained associated with the outcome at a 20% significance level were initially selected and re-analyzed until only the variables that remained associated with the outcome at a 5% significance level remained in the model. The resulting variables from each block were then evaluated in a new regression analysis in which those that remained significantly associated with the outcome at a 5% significance level were selected to calculate the risk of a SMO.

## Results

**Fig. 1:**
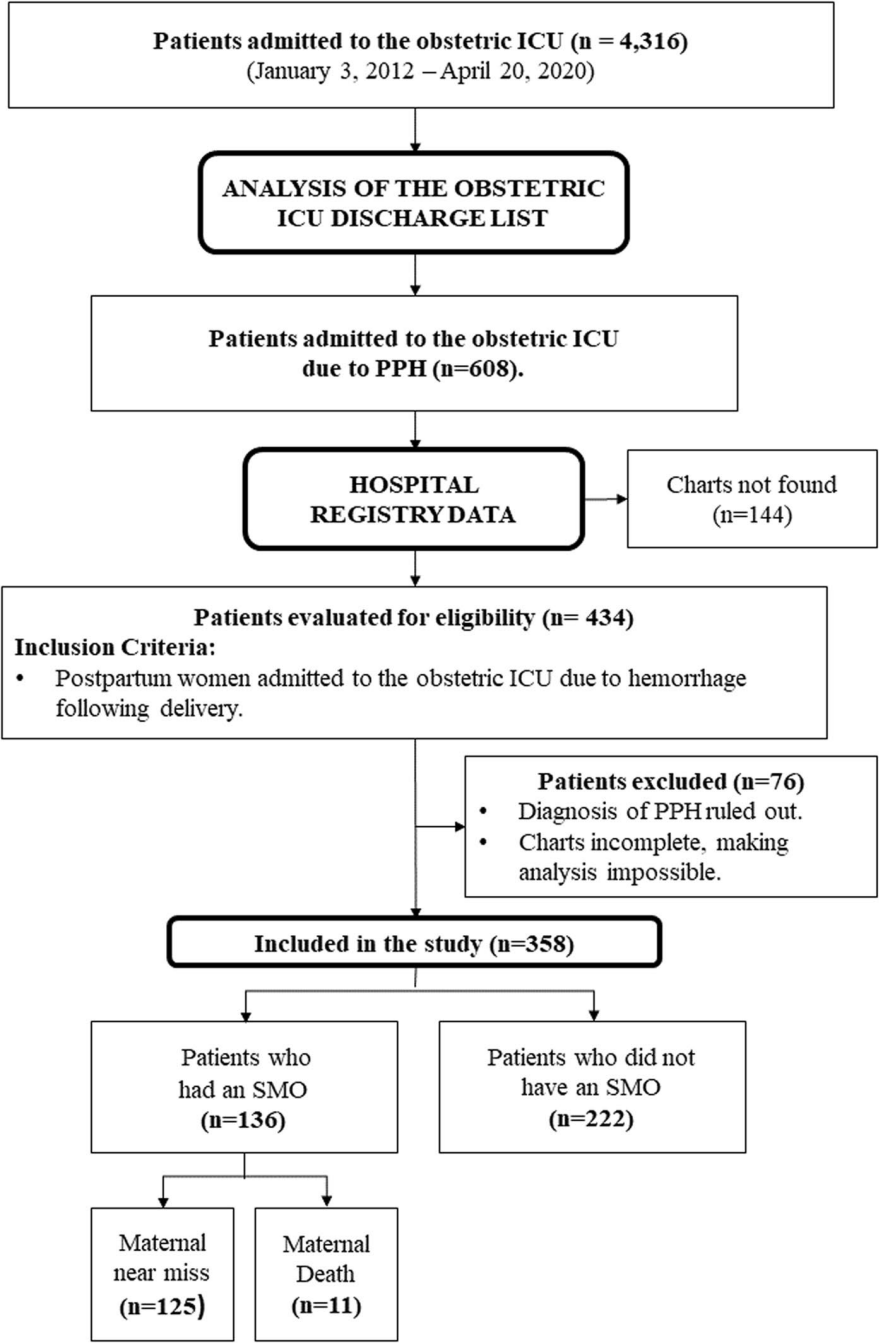
Flowchart of the admission of patients to the study. ICU: intensive care unit; PPH: postpartum hemorrhage; SMO: severe maternal outcome

Between January 2012 and March 2020, 4,316 patients were admitted to IMIP’s obstetric ICU, 608 (14.1%) due to PPH according to the admission records. The files for 434 of these patients were retrievable; however, of these, 76 were excluded either because their diagnosis of PPH was ruled out after their hospital charts had been examined, or because their records were insufficient for analysis. After checking for duplicate files, data were obtained for 358 women. Overall, 136 cases of SMO were identified (37.9%), with 125 women (34.9%) having had an MNM and 11 (3.0%) dying (Fig. 1). The age, parity, delivery route, associated factors and hemorrhage cause of those deaths are tabulated (Table 1). In addition, there were 13 intrauterine fetal deaths (3.6%) (data not shown as tables).

**Table 1:** Clinical characteristics of the maternal deaths subsequent of postpartum hemorrhage

	**Age (years)**	**Parity**	**Delivery**	**Associated factors**	**Cause of hemorrhage**
1	46	1	Cesarean	Hypertension (HELLP syndrome) 1st child after 40 Abruption	Retained placenta
2	31	2	Cesarean	Abnormal placentation	Atony
3	27	2	Vaginal	Induction of labor	Atony
4	17	6	Vaginal	Multipara Episiotomy	Atony, followed by coagulopathy
5	30	2	Vaginal	Hypertension Fetal demise Previous coagulation disorders	Atony and coagulopathy
6	20	2	Cesarean	Hypertension	Atony
7	18	1	Vaginal	Hypertension Uterine distension (polidramnious) Instrumental delivery	Laceration
8	25	3	Vaginal	Vaginal laceration	Atony, retained placenta and laceration
9	33	1	Cesarean	Hypertension	Atony
10	28	1	Vaginal	Abruption	Atony and laceration
11	33	3	Cesarean	Anterior placenta Transfusion-related acute lung injury	Atony, retained placenta, followed by coagulophaty

Of these 358 postpartum women, 245 (68.4%) had delivered in the maternity ward at IMIP, while 113 (31.6%) had been referred from other maternity hospitals in Recife and its greater metropolitan region. Information on the mode of delivery was present on 349 of the records, with 232 patients (66.5%) having been submitted to a Cesarean section (Table 2).

**Table 2:** Sociodemographic and clinical characteristics of the patients admitted to IMIP’s ICU with postpartum hemorrhage

Characteristic	SMO (*n* = 136)	No SMO (*n* = 222)	RR	95%CI	*p*-value
Age (years) (mean/SD)	28.2 (7.4)	25.8 (7.6)	-	-	0.003
Age ≤ 19 years (n/%)	19 (13.9)	51 (22.9)	0.66	0.44–1.00	0.03
Age > 35 years (n/%)	28 (19.8)	28 (12.6)	1.36	1.00–1.86	0.06
Number of pregnancies (median/IQR) ^a^	2 (1–3)	2 (1–3)	-	-	0.0009*
Number of deliveries (median/IQR) ^b^	2 (1–3)	1 (0–2)	-	-	0.0001*
Previous parity ≥ 2 (n/%) ^b^	44 (35.2)	33 (16.2)	1.75	1.34–2.27	0.0001
Gestational age at delivery (median/IQR)	36.6 (32.4–38.5)	37.1 (34.2–39.0)	-	-	0.08*
Gestational age ≤ 34 weeks (n/%) ^c^	30 (36.1)	39 (23.6)	1.46	1.03–2.08	0.04
Gestational age ≥ 41 weeks (n/%) ^c^	2 (2.3)	8 (4.9)	0.56	0.16–1.98	0.5**
Having come from another town or state (n/%)	62 (50.8)	102 (49.0)	1.04	0.78–1.38	0.75
Delivered elsewhere (n/%)	56 (41.2)	57 (25.6)	1.51	1.17–1.96	0.002
No steady partner (n/%) ^d^	18 (24.0)	58 (37.2)	0.64	0.40–1.01	0.04
Education ≤ 8 years of schooling (n/%) ^e^	31 (46.9)	70 (47.3)	0.99	0.66–1.48	0.96
Black- or brown-skinned (n/%) ^f^	41 (77.4)	62 (75.0)	1.08	0.64–1.81	0.75
Prenatal visits (median/IQR) ^g^	6 (4–7)	6 (4–8)	-	-	0.95
Prenatal visits < 6 (n/%) ^g^	25 (51.0)	51 (46.8)	1.12	0.70–1.79	0.62
Gestational age at delivery (median/IQR) ^h^	36.6 (32.4–38.5)	37.1 (34.2–39)	-	-	0.08*
Cesarean section at current pregnancy (n/%) ^i^	91 (68.7)	142 (65.1)	1.10	0.82–1.48	0.49
Previous Cesarean section (n/%) ^j^	34 (60.7)	61 (62.2)	0.95	0.62–1.47	0.85
Previous history of PPH (n/%)	3 (2.2)	3 (1.35)	1.32	0.58–2.97	0.67**
Induction/acceleration of labor	8 (5.8)	32 (14.4)	0.49	0.26–0.93	0.012
Fetal weight (mean/SD)	2514 (1023)	2674 (892)	-	-	0.22
Fetal weight > 4000 g (n/%)	5 (6.8)	7 (4.3)	1.36	0.67–2.74	0.52**
Chorioamnionitis (n/%)	3 (2.2)	9 (4.05)	0.65	0.24–1.75	0.54**
Hypertension/pregnancy-associated hypertension (n/%)	49 (36.0)	129 (58.1)	0.56	0.43–0.75	< 0.01
Previous coagulation disorders (n/%)	6 (4.4)	6 (2.7)	1.33	0.79–2.38	0.38**
Uterine distension (n/%)	5 (3.7)	7 (3.1)	1.10	0.55–2.17	0.77**
Abnormal placentation (n/%)	8 (5.8)	5 (2.3)	1.65	1.05–2.60	0.08**

*IMIP* Instituto de Medicina Integral Prof. Fernando Figueira, *ICU* intensive care unit, *SMO* severe maternal outcome, *RR* risk ratios, *95% CI* 95% confidence interval, *SD* standard deviation, *IQR* interquartile range

^*^Mann–Whitney

^**^Fisher exact test. Number of charts with available data

^a^ 357

^b^ 323

^c^ 248

^d^ 231

^e^ 214

^f^ 141

^g^ 158

^h^ 260

^i^ 349

^j^ 154

The mean age of the patients who had a SMO was 28.2 years compared to 25.8 years for those who did not. In the bivariate analysis, age < 19 years proved to be a protective factor against SMO (RR = 0.66; 95% CI: 0.44–1.00; *p* = 0.03). Women over 35 years of age had similar risk of SMO compared to younger women (RR = 1.36; 95% CI: 1.00–1.86; *p* = 0.06). The two groups were similar in relation to skin color and education (Table 2).

The number of pregnancies was similar in both groups; however, the patients who had had more than two previous deliveries were at a greater risk of having a SMO (RR = 1.75; 95% CI: 1.34–2.27; *p* = 0.0001). Having delivered elsewhere and then being transferred postpartum to IMIP’s obstetric ICU was a significant risk factor for a SMO (RR = 1.51; 95% CI: 1.71–1.96; *p* = 0.002). There was no difference between the groups in relation to the number of prenatal visits attended. Induction or augmentation of labor proved to be a protective factor against SMO (RR = 0.49; 95% CI: 0.26–0.93; *p* = 0.012), while no statistically significant association was found between having delivered by Cesarean section in the current pregnancy and the outcome. There were more cases of premature delivery in the group of patients who had a SMO and the mean birthweight of infants in this group was lower (Table 2).

There was no difference between the two groups regarding the presence of arterial hypertension, abnormal placentation, previous Cesarean section, PPH during a previous pregnancy, previous coagulation disorders, uterine distension or chorioamnionitis (Table 2).

Of the 358 patients admitted due to PPH, 261 women (72.9%) had a diagnosis of uterine hypotonia, 52 (14.5%) of retained products of conception, 48 (13.3%) had bleeding due to lesions of the perineum or during a Cesarean section, and 12 (3.3%) were diagnosed with disorders of the coagulation cascade, including primary coagulopathies and coagulopathies secondary to other conditions such as placental abruption, sepsis, and acute fatty liver of pregnancy (Table 3).

**Table 3:** Cause of postpartum hemorrhage in patients admitted to the ICU according to SMO cases

Cause of hemorrhage	SMO (*n* = 136)	No SMO (*n* = 222)	RR	95% CI	*p*-value
**Tone** (n/%)	98 (72.0)	163 (73.4)	0.95	0.71–1.28	0.77
**Tissue** (n/%)	24 (17.6)	28 (12.6)	1.26	0.90–1.75	0.19
**Trauma** (n/%)	17 (12.5)	31 (13.9)	0.92	0.61–1.38	0.69
**Thrombin** (n/%)	6 (4.41)	6 (2.7)	1.33	0.74–2.38	0.38

*ICU* intensive care unit, *SMO* severe maternal outcome, *RR* risk ratios, *95% CI* 95% confidence interval

In the hierarchical multiple logistic regression controlling for potential confounders, the factors that remained associated with an SMO were: gestational age ≤ 34 weeks (adjusted odds ratio [AOR] = 2.01; 95% CI: 1.12–3.64; *p* = 0.02), previous parity ≥ 2 (AOR = 2.20; 95% CI: 1.10–4.68; *p* = 0.02) and having delivered in another maternity hospital (AOR = 2.22; 95% CI: 1.02–4.81; *p* = 0.04) (Table 4). This model correctly predicted 70.2% of the cases and the area under the receiver operating characteristic (ROC) curve (AUC) was 62%, with a 95% CI of 0.56 to 0.68.

**Table 4:** Multivariate analysis of conditions associated with SMO

Factor	Coefficient	Standard Error	Adjusted Odds Ratio	95%CI	*p*-value
Gestational age ≤ 34 weeks	0.70	0.30	2.02	1.12–3.64	0.02
Previous parity ≥ 2	0.82	0.37	2.28	1.11- 4.69	0.025
Not having delivered in the institute	0.80	0.39	2.22	1.03- 4.81	0.043
Constant	-1.15	0.19	-	-	< 0.0001

*SMO* severe maternal outcome, *PPH* postpartum hemorrhage, *ICU* intensive care unit, *95% CI* 95% confidence interval. This model correctly predicted 70.2% of the cases and the area under the receiver operating characteristic (ROC) curve (AUC) was 62% (95% CI: 0.56–0.68)

## Discussion

Maternal bleeding remains one of the principal causes of morbidity and mortality in pregnant and postpartum women in Brazil. Therefore, determining the specific factors associated with SMO (MNM and maternal death) resulting from PPH is vital when proposing improvements to protocols for the prevention and treatment of this complication. In the present cohort, there were 136 cases of SMO (37.9%): 125 women (34.9%) who had an MNM and 11 (3.0%) who died. In addition, there were 13 cases of intrauterine fetal death (3.6%). The etiologies of PPH follow trends reported in other studies [9, 19], with the principal cause being tone (72.9%) followed by tissue (14.5%), trauma (13.3%) and thrombin (3.3%).

There was no difference between the two groups in relation to place of residence, skin color, education, access to prenatal care or any other socioeconomic factors and the development of a SMO. Nevertheless, studies have shown that Black women are at a greater risk of PPH and SMO, with this probably being related more to sociodemographic inequities in access to health care and obstetric care rather than to any factors directly related to skin color [3, 20, 21]. Furthermore, the present study was conducted in an exclusively public hospital, which, in itself, implies in a lower income and schooling segment of Brazilian society. Data relating to these characteristics were found to be missing on some of the hospital records, which could have negatively affected the analysis and is, in itself, a problem in the sense that failing to record skin color is to render the problem invisible, reflecting the deep structural racism in Brazilian society that is equally present in the healthcare model [20, 22]. The nationwide “*Childbirth in Brazil*” study, involving 266 institutes throughout the entire country, showed that patients with lower incomes, the population that relies on the public healthcare service in Brazil, are at a greater risk of developing a SMO [17].

PPH is a complication that is difficult to predict, and prevention and prognosis mainly depend on inpatient management, which should follow a proven sequence of pharmacological and/or surgical interventions until the condition is resolved [23–25]. Although the risk factors for PPH are well known, it is difficult to determine what leads to severe PPH and to SMO due to hemorrhage, since patients with no risk factors can present with severe PPH, while the majority of high-risk patients have no significant blood loss, with the risk of severe hemorrhage in this group ranging from 2 to 7% [26–29].

Mean maternal age was greater in the group of women who had a SMO (28.2 years in the SMO group and 25.8 years in the group of women who did not have a SMO; *p* = 0.003). Older women are known to be more likely to be multiparous and to have comorbidities in addition to the natural metabolic and physical alterations of aging [29–32], all of which increase not only their likelihood of PPH but also of progression to a SMO. In support of this hypothesis, in the multivariate analysis, previous parity ≥ 2 was associated with a greater risk of SMO (RR = 1.75; 95% CI: 1.34–2.27; *p* = 0.0001); however, no association was found with maternal age in the multivariate analysis, probably due to other overlapping factors such as, indeed, parity.

Contradicting initial expectations that arterial hypertension would increase the risk of severe PPH [33, 34], no association was found with SMO either in the bivariate analysis or in the logistic regression analysis. Since only patients who had been admitted to the ICU were evaluated, hypertension may have been a confounding factor. In fact, in the institute in which the study was conducted, it is common for patients with hypertensive disorders such as severe preeclampsia to be admitted to the ICU for monitoring, for administration of magnesium sulfate or for the management of severe hypertension, with the associated PPH perhaps being mild and without any repercussions. This could be why no association was found between the increase in arterial blood pressure and a SMO.

Median gestational age at delivery was 36.6 weeks in the patients who had a SMO compared to 37.1 weeks for those who did not. Delivery at less than 34 weeks was associated with a SMO both in the bivariate analysis (OR = 1.46; 95% CI: 1.03–2.08; *p* = 0.04) and following logistic regression analysis (OR = 2.01; 95% CI: 1.12–3.64; *p* = 0.02). This finding may be related to the higher rate of prematurity in cases of severe preeclampsia, HELLP syndrome and placental abruption, which are risk factors for PPH and are often indications for premature delivery [34–37]. The presence of retained placenta due to incomplete delivery of the placenta is also more common in premature deliveries, increasing the risk of PPH due to retained tissue [37, 38]. Furthermore, there are hypotheses that drugs such as magnesium sulfate and the tocolytics used in the management of premature delivery could lead to a greater incidence of PPH [39, 40]. These drugs, however, were not specifically investigated in this study.

Although having had a previous Cesarean section or having undergone a Cesarean section in the current pregnancy are factors classically associated with a greater risk of SMO [24, 25, 41], in the present study this association could not be established, probably because the number of women in the study was insufficient for it to be identified. In Brazil, where cesarean sections are highly predominant, the lack of clarity regarding the indication for Cesarean sections on the hospital charts is a problem itself and could also have hampered this evaluation, since in some of these cases it was impossible to identify whether the Cesarean section performed was elective or clinically indicated. Some studies have reported an important difference in the risk of PPH depending on whether the Cesarean section was elective or intrapartum [42, 43].

There were only 13 cases of abnormal placentation: 8 (5.8%) in patients who had a SMO and 5 (2.3%) in those who did not. This suggests a tendency for this clinical situation to be a risk factor for SMO; however, since there were so few cases of this complication in the study, the statistically significant association found in other studies could not be confirmed [27, 44]. Nevertheless, in a cohort study of 269 women submitted to a scheduled Cesarean section, abnormal placentation was associated with 72% of hysterectomies and 49% of ICU admissions, highlighting the magnitude of the association between this condition and SMO [45]. Likewise, in other less prevalent conditions such as uterine distension and chorioamnionitis, conditions present in only 12 patients each in the present sample, no statistically significant association was found with the development of a SMO.

In the present sample, having given birth elsewhere and then having been transferred to this ICU constituted a risk factor for the development of an SMO (RR = 1.51; 95% CI: 1.17–1.96; *p* = 0.002) and this association persisted in the multivariate analysis (RR = 2.22; 95% CI: 1.02–4.81; *p* = 0.04). This corroborates the findings of other studies showing that time and logistic issues in accessing adequate health facilities are crucial factors in avoiding PPH [16, 46]. Delayed and inadequate transfers seriously impact patients’ prognosis. Therefore, the Pan American Health Organization and WHO’s strategies such as the *Zero Maternal Deaths from Hemorrhage* initiative are crucial in low-income countries to optimize the flow and care provided during the transfer of critically ill patients [47]. No data were found on the hospital charts regarding the use of anti-shock garments, devices that are still largely unavailable in Brazil; cases of patients transferred following surgery to control damage; the time between the diagnosis of PPH and the patient’s arrival at the ICU; or any other relevant data on the quality of patient transfers [47].

Finally, the use of methods for inducing/accelerating labor constituted a protective factor against SMO (RR = 0.49; 95% CI: 0.26–0.93; *p* = 0.012) in the bivariate analysis; however, this association did not persist in the multivariate analysis. The association could have been due to confounding factors alone; however, it could be explained by the fact that, although induction of labor increases the likelihood of PPH, generally in this situation the etiology is uterine atony. Since this is the most common cause of PPH, it is also the cause that is simplest to control, either because of the wide availability of drugs that promote uterine contractility or the fact that the institutional bundles and training on maternal hemorrhage focus primarily on this cause [48, 49]. Furthermore, on many of the hospital charts referring to patients transferred from other healthcare services, no details on the history of childbirth were found, making it impossible to adequately evaluate the association between induction and PPH due to the omission of this information.

Few studies have been conducted in Brazil to evaluate women with a diagnosis of PPH who went on to have a SMO. This study provided the opportunity to select cases that were already in an ICU, i.e. their condition was already more severe and there was a greater likelihood of an unfavorable outcome. Nevertheless, in retrospective studies, the risk of missing interesting data that the healthcare team could have provided on the chart represents an important limitation; consequently, certain analyses could not be carried out and associations that might have been present could not be demonstrated.

The strongpoint of the study was the evaluation of a large volume of patients who had an unfavorable outcome; therefore, even with the loss of data that decreased the final sample size and limited the associations between some of the variables evaluated, it was possible to pinpoint important factors associated with SMO, principally previous parity ≥ 2, gestational age ≤ 34 weeks and not having given birth in the institute.

## Conclusion

Women who have a SMO often have several overlapping comorbidities and clinical characteristics. Nevertheless, in this study, previous parity ≥ 2, gestational age ≤ 34 weeks and not having given birth in the institute were factors associated with a poor prognosis in PPH, with a greater risk of SMO. Giving due importance to these factors will allow attending physicians and healthcare managers to improve clinical practice and intra- and inter-hospital transfers.

### Supplementary Information

**Additional file 1.**

## Data Availability

The datasets used and analyzed during the current study are available from the corresponding author upon reasonable request.

## Abbreviations

95% CI: 95% Confidence intervals
AUC: Area under the curve
ICU: Intensive care unit
IMIP: Instituto de Medicina Integral Prof. Fernando Figueira
MNM: Maternal near miss
PPH: Postpartum hemorrhage
ROC: Receiver operating characteristic curve
RR: Risk ratios
SMO: Severe maternal outcome
WHO: World Health Organization
