# Supplementary material for: Factors associated with severe maternal outcome in patients admitted to an intensive care unit in northeastern Brazil with postpartum hemorrhage: a retrospective cohort study
Source: BMC Pregnancy Childbirth. 2023 Aug 10;23:573. doi: 10.1186/s12884-023-05874-1 (PMC10413525; doi:10.1186/s12884-023-05874-1)
Supplement: Supplementary file 1 — Additional file 1. [file 12884_2023_5874_MOESM1_ESM.doc]

| **Stage 0: Every delivery – Prevention and Recognition of Postpartum Bleeding**  **Prenatal Assessment & Planning** | | |
| --- | --- | --- |
| **Identify and prepare for patients in special situations: placenta previa/accreta, coagulopathy, who reject blood products (Jehovah's Witnesses).**  **Identify and treat severe anemia: If oral iron fails, treat with parenteral iron until target Hb/Ht is achieved** | | |
| **EVALUATION AND PLANNING ON ADMISSION** | | **CONTINUED RISK ASSESSMENT** |
| Check blood type and s  Indirect Coombs (IC) in prenatal care  If not available:  □ Request blood typing and IC  If IC positive (except low titers due to immunoglobulin use):  □ Cross-match and reserve 2U PRBCs | □ Assess risk factors on admission, during labor and in the puerperium and at each team change  If medium risk:  □ Reserve blood sample and forward to TA  □ Review bleeding protocol  If high risk:  □ Request cross-matching and reservation of 2U PRBCs  □ Review bleeding protocol  □ Notify anesthesiologist  □ Ensure high caliber access (18G or greater) | **In labor:**  □ Second extended period  □ Prolonged use of oxytocin  □ Active genital bleeding  □ Chorioamnionitis  □ Use of magnesium sulfate  □ Consider multiple risk factors as High Risk  □ Monitor increased postpartum bleeding |
| **RISK ASSESSMENT FOR HEMORRHAGE ON ADMISSION** | | |
| □ **Low Risk (continue risk assessment)** | □ **Medium Risk (book blood sample and**  **forward to TA and continue risk assessment)** | □ **High Risk (typing and IC - research of**  **irregular antibodies)** |
| No previous uterine incision  singleton pregnancy  ≤ 4 previous vaginal deliveries  No known clotting disorders  No PPH history | Cesarean(s) or previous uterine surgery(s)  Uterine hyperdistension (twins, polyhydramnios, macrosomia)  > 4 previous vaginal deliveries  Chorioamnionitis  Previous history of PPH  Large uterine fibroids  Hypertension disorders  Obesity (BMI > 35) | 2 or more medium risk situations  PE with severity criteria  Abnormal placentation  Hematocrit < 30%  Active bleeding on admission (exclude tampon)  Platelets < 100,000  Coagulopathy or use of anticoagulants  Placental abruption |
| **EVERY DELIVERY – PROPHYLACTIC OCYTOCIN, QUANTIFICATION OF BLOOD LOSS AND STRICT MONITORING OF**  **VITAL DATA** | | |
| After all vaginal deliveries: Oxytocin: 10 U IM (vaginal delivery);  All cesarean sections: Tranexamic acid 1 g IV over 5 minutes, 10 minutes before incision  If cesarean section, “rule of 3” after fetal extraction and after completing surgery run 20UI in 4h  Blood loss assessment: 15 in 15' for 2 hours and then every 4 hours (cesarean section); 6 in 6 hours (vaginal delivery)  Monitoring of Vital Data: 15 in 15' for 2 hours and then every 4 hours (cesarean section); 6 in 6 hours (vaginal delivery) | | |
| **If EBL > 500 mL for Vaginal Delivery OR > 1000 mL for Cesarean Section OR HR ≥ 110 Bpm, BP ≤ 85/45 mmHg, SpO2 < 95% AND Increased Bleeding OR Shock Index (HR/SBP) between 1.0 and 1, 3 go to Stage 1** | | |

;;;

| **STAGE 1: QBL > 500 ML FOR VAGINAL DELIVERY OR > 1000 ML FOR CESAREAN OR HR ≥ 110 BPM, BP ≤ 85/45 MMHG, SPO2 < 95% OR SHOCK INDEX (HR/SBP) BETWEEN 1.0 AND 1.3 WITH INCREASED BLEEDING** | | |
| --- | --- | --- |
| **MOBILIZE** | **ACT** | **THINK** |
| □ Enable protocol and start checklist  □ Call Obstetrician  □ Call another Nurse/midwife  □ Alert Anaesthesiologist  □ Communicate TA  □ Delegate functions  □ Reserve 2U PRBCs | □ Provide 2 venous accesses at least 16G;  □ Draw blood (HMG/PLQS, COAG, Fibrinogen) and clot test;  □ Start volemic replacement with free SF;  □ Perform vigorous uterine massage;  □ Assess airway and breathing and start O2 to maintain SpO2 > 95%;  □ Empty bladder;  □ Monitor pulse, BP, SpO2 15'/15';  □ Weigh material and calculate PS every 15 min;  □ Keep patient warm.  □ Administer 1.0 g of IV tranexamic acid over 10 min. and Start 20 IU of oxytocin/500 ml SF in free infusion concomitantly;  □ Administer methylergometrine IM 0.25 mg if bleeding continues after rapid administration of oxytocin.  □ Perform birth canal review;  □ Assess the causes (4 Ts – on the right);  □ Administer misoprostol 600 mcg SL or 800 mcg PO if bleeding continues after administering methylergometrine and proceed to stage 2 management.  **If cesarean section and open abdomen**  □ Inspect source of bleeding at all levels: broad ligament, posterior uterus, placenta accreta, etc. | **Etiology:**  **Tone**  **Trauma**  • Lacerations of the birth canal  • Bruises  • Uterine rupture, broad ligament haematoma, uterine inversion.  **Tissue**  • Retained products (placental remains, membranes, clots)  • Placenta accreta  **Thrombin**  • Treat underlying diseases and assess coagulation serially  • Replace blood components  • If positive clot test starts FFP (no lab)  If the patient stabilizes and bleeding is controlled in stage 1, maintain strict postpartum surveillance. If Trauma, Tissue and/or Thrombin present proceed to stage 2 |
| **If QBL > 1000 ml and < 1500 ml and continued bleeding or unstable vitals OR Shock Index (HR/SBP) between 1.4 and 1.6**  **move to Stage 2** | | |

| **STAGE 2: QBL > 1000 ML AND < 1500 ML, CONTINUED BLEEDING OR UNSTABLE VITAL DATA OR SHOCK INDEX (HR/SBP) BETWEEN 1.4 AND 1.6** | | |
| --- | --- | --- |
| **MOBILIZE** | **ACT** | **THINK** |
| □ Communicate anesthesiologist;  □ Request 2U of PRBCs;  □ Reserve 2U of FFP and 1U of PLQ;  □ Notify TA, Designate person responsible for transporting material for exams and blood products;  □ Assign nurses and/or midwifes to perform the actions in the right column;  □ Communicate with TA, log events, complete checklist;  □ Call SP to communicate with the family.  □ Use standardized communication  **SOCIAL WORKER OR PSYCHOLOGIST**  □ Communicate appropriately and sensitively with the family | **OBSTETRICIAN**  □ Administer 1.0 g of IV tranexamic acid in 10 min if bleeding persists after 30 minutes of the first dose;  □ Maintain oxytocin 20 IU/500 ml SF 125 mL/h and fluids;  □ Abdominal or vaginal/abdominal uterine massage (figures 1 and 2);  □ Progress to other interventions. WITHOUT DELAY. See right column;  □ Transfuse 2U PRBCs, based on response and clinical signs, (INDEPENDENT OF LABORATORY RESULTS);  □ Consider PRBCs O- if CP not available Request transfer to OR;  □ Request complementary tests (HMG/PLQ, COAG, Fibrinogen, GASO, ions, lactate).  **NURSE/MIDWIFE**  □ Establish 2 venous accesses at least 16 G, if not yet available Provide blood equipment and solution warmers;  □ Administer medications, blood products and collect blood for tests Keep patient warm.  □ Monitor and report DVs every 5-10 minutes;  □ Quantify accumulated blood loss every 5-15 minutes Perform bladder catheterization with a volume measuring bag;  □ Transfer patient to OR and call anaesthetist;  □ Prepare room and material for surgery.  **ANESTHESIOLOGIST**  □ Thaw two units of PFC if transfused > 2U PRBCs;  □ Provide platelets;  □ Prepare to activate massive bleeding protocol. | **Tonus:** Intrauterine balloon. If cesarean: Compressive sutures (B-Lynch, Hayman, Cho, Barbosa da Silva).  **Trauma:** Repair cervical and vaginal lacerations; investigate and treat uterine rupture, broad ligament hematoma, uterine inversion.  **Tissue:** Placental remains, acretism (healing, curettage). If bleeding persists in the presence of accreta, refer early for surgical treatment and move on to stage 3.  **Thrombin:** Continue serial coagulation assessment and replace blood components.  If the bleeding stops and the patient stabilizes, refer him to ICU and maintain surveillance for at least 12 hours.  **Maintain oxytocin infusion 20 IU/500 ml of SF at 67.5 ml/h for up to 24 hours, with monitoring of water intoxication.** |
| **If accumulated QBL > 1500 ml, OR > 2 units of RBCs transfused OR unstable vitals OR suspected DIC**  **(disseminated intravascular coagulation) OR Shock Index (HR / SBP) ≥ 1.7 proceed to stage 3 management** | | |

| **Stage 3: PSQ > 1500 mL OR > 2 Units of RBCs Transfused OR Vital Data Unstable OR Suspected DIC (Disseminated Intravascular Coagulation) OR Shock Index (HR/SBP) ≥ 1.7** | | |
| --- | --- | --- |
| **MOBILIZE** | **ACT** | **THINK** |
| □ Enable Massive Bleeding protocol  □ Call Intensivist doctor;  □ Reassess available personnel and request help if needed;  □ Request transfer to the OR if not already performed  □ Start preparations for postpartum transfer to ICU  □ Communicate appropriately and sensitively with the family | **OBSTETRICIAN**  □ Order massive hemorrhage package (PRBCs, FFP, PLQ);  □ Switch to OR if not already done;  □ Repeat complementary tests (HMG/PLQ, COAG, Fibrinogen, GASO, ions, lactate) every 30-60 min.  **ANESTHESIOLOGIST**  □ Start vasopressor support if necessary;  □ Perform endotracheal intubation if necessary;  □ Establish central venous access if necessary;  □ Establish line for intra-arterial pressure;  □ Replace calcium if hypocalcemia;  □ Monitor electrolytes.  **NURSE**  □ Administering medication, blood products and collecting blood for tests;  □ Warm solutions to be infused. Provide quick infuser for fluids;  □ Keep patient warm;  □ Apply compression stockings to the lower limbs.  □ Announce vital data and accumulated blood loss every 5-10 min;  □ Prepare room and material for surgery if not yet done.  **MIDWIFE**  □ Continue event recording and complete checklist  □ Assist and coordinate nurses | Interventions based on etiology (Tone, Trauma, Tissue, Thrombin) not yet performed  Prevent hypothermia and acidemia Conservative or definitive surgery:  • Progressive devascularization (uterine, ovarian, hypogastric ligation)  • Compressive sutures (B-Lynch, Barbosa da Silva, Hayman)  • Hysterectomy  For Resuscitation: Aggressive transfusion based on vital data and blood loss  After 2 U CHM Almost equal PFC/CHM ratios (4-6 PRBCs / 4 FFP / 1 PLQ)  **Coagulopathy**  Replace clotting factors to maintain: Platelets above 50,000/ml  Fibrinogen ≥ 200mg/dL  INR < 1.5 |

#### SUBTITLES

####

#### AC = Rooming-in

#### TA = Transfusion Agency

#### OR = Operating Room

#### PRBC = Packed Red Blood Cells

#### CI = Indirect Coombs

#### COAG = Coagulogram

#### IV = Intravenous

#### GASO = Arterial Gasometry

#### BG = Blood Group

#### HMG = Haemogram

#### IM = Intramuscular

#### CP = Cross Proof

#### FFP = Fresh Frozen Plasma

#### PLQ = Platelets

#### EBL = Estimated Blood Loss (visual assessment)

#### QBL = Quantified Blood Loss (quantitative assessment)

#### SS = Saline Solution

#### SL = Sublingual

#### DR = Delivery Room

#### PPH = Postpartum haemorrhage

#### ICU = Intensive Care Unit
